# Using and interpreting cost-effectiveness acceptability curves: an example using data from a trial of management strategies for atrial fibrillation

**DOI:** 10.1186/1472-6963-6-52

**Published:** 2006-04-19

**Authors:** Elisabeth Fenwick, Deborah A Marshall, Adrian R Levy, Graham Nichol

**Affiliations:** 1 Public Health and Health Policy, University of Glasgow, Glasgow, UK; 2 Department of Clinical Epidemiology and Biostatistics, McMaster University and Centre for Evaluation of Medicines, St. Joseph's Hospital, Hamilton, ON, Canada; 3 Department of Health Care and Epidemiology, University of British Columbia, Vancouver, BC, Canada; 4 Harborview Center for Prehospital Emergency Care, University of Washington, Seattle, USA

## Abstract

**Background:**

The cost-effectiveness acceptability curve (CEAC) is a method for summarizing the uncertainty in estimates of cost-effectiveness. The CEAC, derived from the joint distribution of costs and effects, illustrates the (Bayesian) probability that the data are consistent with a true cost-effectiveness ratio falling below a specified ceiling ratio. The objective of the paper is to illustrate how to construct and interpret a CEAC.

**Methods:**

A retrospective cost-effectiveness analysis of the Atrial Fibrillation Follow-up Investigation of Rhythm Management (AFFIRM) randomized controlled trial with 4060 patients followed for 3.5 years. The target population was patients with atrial fibrillation who were 65 years of age or had other risk factors for stroke or death similar to those enrolled in AFFIRM. The intervention involved the management of patients with atrial fibrillation with antiarrhythmic drugs (rhythm-control) compared with drugs that control heart rate (rate-control). Measurements of mean survival, mean costs and incremental cost-effectiveness were made. The uncertainty surrounding the estimates of cost-effectiveness was illustrated through a cost-effectiveness acceptability curve.

**Results:**

The base case point estimate for the difference in effects and costs between rate and rhythm-control is 0.08 years (95% CI: -0.1 years to 0.24 years) and -US$5,077 (95% CI: -$1,100 to -$11,006). The CEAC shows that the decision uncertainty surrounding the adoption of rate-control strategies is less than 1.7% regardless of the maximum acceptable ceiling ratio. Thus, there is very little uncertainty surrounding the decision to adopt rate-control compared to rhythm-control for patients with atrial fibrillation from a resource point of view.

**Conclusion:**

The CEAC is straightforward to calculate, construct and interpret. The CEAC is useful to a decision maker faced with the choice of whether or not to adopt a technology because it provides a measure of the decision uncertainty surrounding the choice.

## Background

Health care decision-makers need to choose whether or not to reimburse health technologies, and cost-effectiveness is increasingly one of the criteria used to guide that choice. Estimates of cost-effectiveness are inevitably associated with some degree of uncertainty, generated jointly from the estimate of effectiveness and costs. In addition to the point estimate for cost-effectiveness, a confidence interval around the estimate provides the decision-maker with a measure of the degree of uncertainty. The cost-effectiveness acceptability curve (CEAC) is an intuitive graphical method of summarizing information on uncertainty in cost-effectiveness estimates. The CEAC is straightforward to construct and interpret, which is why it is increasingly becoming a part of economic evaluations for decision makers.

### Objective

As the use of CEACs becomes more widespread [1-11] it is important that researchers learn how to construct, apply, and interpret these curves. In this paper, a recently published cost-effectiveness analysis comparing rhythm-control versus rate-control treatment for atrial fibrillation is used to illustrate the CEAC [12]. After a brief introduction on cost-effectiveness analysis and uncertainty, to frame the issue, this paper describes how to construct a CEAC and how it should be interpreted. This paper concentrates specifically on decisions involving two interventions. For evaluations comparing more than two interventions see Fenwick *et al*., 2001 [13].

### The AFFIRM study

The objective of the Atrial Fibrillation Follow-up Investigation of Rhythm Management (AFFIRM) trial was to compare the effectiveness of rhythm-control versus rate-control in patients with atrial fibrillation [14]. After a mean follow-up time of 3.5 years in both arms of the trial, there was a non-significant mean survival gain of 0.08 years (P = 0.10, based on a two-sided test) for subjects randomized to the rate-control arm. A trial-based economic evaluation was undertaken to estimate the cost-effectiveness of rhythm-control versus rate-control based on the results from AFFIRM [12]. Rate-control subjects consumed fewer resources (hospital days, pacemaker procedures, cardioversions, and short stay and emergency room visits) and had a lower per patient cost than rhythm-control subjects (US $5,077 per person). Non-parametric bootstrapping methods were used to estimate the distribution of incremental costs and effects associated with rhythm-control compared to rate-control. We found that rate-control was less costly and more effective than rhythm-control in 95% of the replicates under a range of cost assumptions. We concluded that rate-control was a cost-effective approach to the management of atrial fibrillation when compared to rhythm-control.

The cost-effectiveness analysis of the AFFIRM trial data provides a good illustration of how uncertainty in the estimate of cost-effectiveness must consider the distribution of both costs and effects. Based on the effectiveness data alone, one might conclude that either treatment is equally effective, with a small and not statistically significant trend in survival favoring one arm (rate-control). After considering the joint distribution of costs and effects, rate-control is the favored initial approach to treatment because of the lower cost compared to rhythm-control, despite the absence of a statistically significant difference in survival between the two treatments.

### Cost-effectiveness analysis

A cost-effectiveness analysis enumerates the additional resources consumed for an improvement in the effects (for example, survival or quality-adjusted life years) associated with one health intervention compared to another. The result can be summarized as an incremental cost-effectiveness ratio (ICER) – a measure of the additional cost per unit of health gain. The underlying calculation for the ICER comparing rate-control versus rhythm-control treatment in patients with atrial fibrillation was:

ICER=Average Costrate-control−Average Costrhythm-controlAverage Effectrate-control−Average Effectrhythm-control
 MathType@MTEF@5@5@+=feaafiart1ev1aaatCvAUfKttLearuWrP9MDH5MBPbIqV92AaeXatLxBI9gBaebbnrfifHhDYfgasaacH8akY=wiFfYdH8Gipec8Eeeu0xXdbba9frFj0=OqFfea0dXdd9vqai=hGuQ8kuc9pgc9s8qqaq=dirpe0xb9q8qiLsFr0=vr0=vr0dc8meaabaqaciaacaGaaeqabaqabeGadaaakeaacqqGjbqscqqGdbWqcqqGfbqrcqqGsbGucqGH9aqpdaWcaaqaaiabbgeabjabbAha2jabbwgaLjabbkhaYjabbggaHjabbEgaNjabbwgaLjabbccaGiabboeadjabb+gaVjabbohaZjabbsha0naaBaaaleaacqqGYbGCcqqGHbqycqqG0baDcqqGLbqzcqqGTaqlcqqGJbWycqqGVbWBcqqGUbGBcqqG0baDcqqGYbGCcqqGVbWBcqqGSbaBaeqaaOGaeyOeI0IaeeyqaeKaeeODayNaeeyzauMaeeOCaiNaeeyyaeMaee4zaCMaeeyzauMaeeiiaaIaee4qamKaee4Ba8Maee4CamNaeeiDaq3aaSbaaSqaaiabbkhaYjabbIgaOjabbMha5jabbsha0jabbIgaOjabb2gaTjabb2caTiabbogaJjabb+gaVjabb6gaUjabbsha0jabbkhaYjabb+gaVjabbYgaSbqabaaakeaacqqGbbqqcqqG2bGDcqqGLbqzcqqGYbGCcqqGHbqycqqGNbWzcqqGLbqzcqqGGaaicqqGfbqrcqqGMbGzcqqGMbGzcqqGLbqzcqqGJbWycqqG0baDdaWgaaWcbaGaeeOCaiNaeeyyaeMaeeiDaqNaeeyzauMaeeyla0Iaee4yamMaee4Ba8MaeeOBa4MaeeiDaqNaeeOCaiNaee4Ba8MaeeiBaWgabeaakiabgkHiTiabbgeabjabbAha2jabbwgaLjabbkhaYjabbggaHjabbEgaNjabbwgaLjabbccaGiabbweafjabbAgaMjabbAgaMjabbwgaLjabbogaJjabbsha0naaBaaaleaacqqGYbGCcqqGObaAcqqG5bqEcqqG0baDcqqGObaAcqqGTbqBcqqGTaqlcqqGJbWycqqGVbWBcqqGUbGBcqqG0baDcqqGYbGCcqqGVbWBcqqGSbaBaeqaaaaaaaa@BCA4@

where costs were measured in US dollars and effects were measured in life years gained.

**Figure 1:**
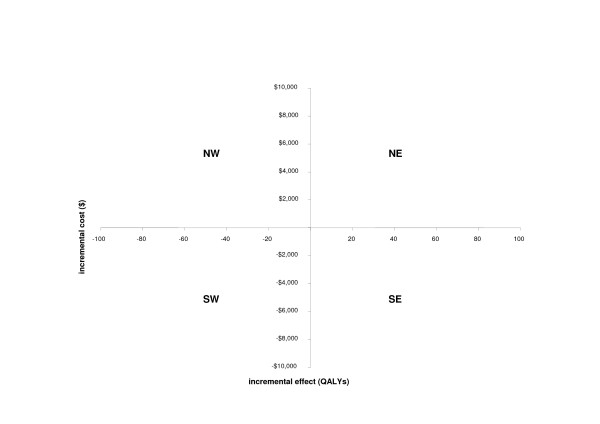
The incremental cost-effectiveness plane. NE = northeast quadrant; NW = northwest quadrant; SE = southeast quadrant; SW = southwest quadrant; QALY = quality adjusted life year

The incremental cost and incremental effect can be represented visually using the incremental cost-effectiveness plane [15]. The horizontal axis divides the plane according to incremental cost (positive above, negative below) and the vertical axis divides the plane according to incremental effect (positive to the right, negative to the left). This divides the incremental cost-effectiveness plane into four quadrants through the origin (Figure 1).

Each quadrant has a different implication for the decision. If the ICER for rate-control compared to rhythm-control fell in the southeast quadrant, with negative costs and positive effects, rate-control would be more effective (better survival) and less costly than rhythm-control (i.e., rate-control 'dominates' rhythm-control). Interventions falling in this quadrant are always considered cost-effective. If the ICER fell in the northwest quadrant, with positive costs and negative effects, rate-control would be more costly and less effective than rhythm-control (i.e., rate-control is 'dominated' by rhythm-control). Interventions falling in this quadrant are never considered cost-effective. If the ICER fell in the northeast quadrant, with positive costs and positive effects, or the southwest quadrant, with negative costs and negative effects, trade-offs between costs and effects would need to be considered. These two quadrants represent the situation where rate-control may be cost-effective compared to rhythm-control, depending upon the value at which the ICER is considered good value for money.

#### Value for money

In order to decide if an intervention offers "good" value for money, the ICER must be compared to a specified monetary threshold. This threshold represents the maximum amount that the decision-maker is willing to pay for health effects (maximum acceptable ceiling ratio). The intervention is deemed cost-effective if the ICER falls below this threshold and not cost-effective otherwise [16]. For example, if a decision-maker is willing to pay $50,000 for a year of life, the intervention is considered cost-effective if the ICER is below $50,000 per life year gained. In situations where a threshold is not stated explicitly, the act of decision-making implies a value for the threshold.

Is rate-control in the management of patients with atrial fibrillation 'good value for money' compared to rhythm-control? The base case cost-effectiveness analysis of the AFFIRM trial reported that rate-control demonstrated a non-significant mean survival gain compared to rhythm-control (0.08 years, CI -0.1 to 0.24), and that rate-control was less costly than rhythm-control (US -$5,077, CI -$1,100 to -$11,006). In this situation, no ICER would be calculated because the point estimate for the ICER falls in the southeast quadrant of the cost-effectiveness plane where rate-control is more effective and less costly than the rhythm-control (i.e., rate-control dominates rhythm-control). Rate-control would clearly be considered 'good value for money'. However, this does not take any uncertainty in the estimates of costs and effects into consideration and decision makers will be interested to ascertain how sure they can be that this is the correct conclusion to make.

#### Uncertainty in costs and effects

Due to imperfect information on the effectiveness of intervention and the resources consumed for treatment, both the costs and effects of health interventions are inevitably associated with some degree of uncertainty. The presence of this uncertainty means that there is inevitably some possibility that decisions made on the basis of the available (uncertain) information will be incorrect and introduces the possibility of error into decision-making. The use of stochastic (e.g., bootstrapping) and probabilistic techniques (e.g., Monte Carlo simulation), for trial analyses and modeling studies respectively, to generate the sampling distribution of the joint mean cost and efficacy has enabled quantification of the uncertainty surrounding the estimates of costs and effects.

**Figure 2:**
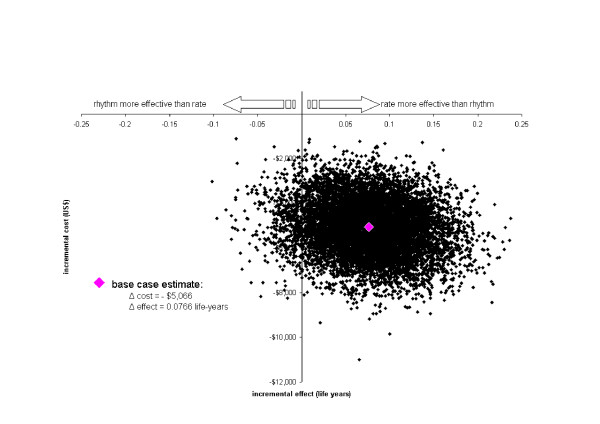
Scatter plot of estimated joint density of incremental costs and incremental effects of rate-control vs. rhythm-control obtained by bootstrap re-sampling in the AFFIRM trial.

One method that is typically used to represent the uncertainty in the costs and effects associated with a technology is a scatter plot of simulated (by bootstrapping or probabilistic modeling) incremental cost and effect pairs on the incremental cost-effectiveness plane [17]. Figure 2 illustrates the incremental cost-effectiveness plane for the cost and effectiveness data bootstrapped from the AFFIRM trial. The base case point estimate for the difference in costs and effects between rate and rhythm-control is marked at 0.08 years and -$5,077. The scatter plot additionally illustrates the uncertainty surrounding the estimates of expected incremental cost (in 2002 US dollars) and expected incremental effect (life years gained) associated with rate-control compared to rhythm-control strategies for the management of patients with atrial fibrillation enrolled in the trial. The location of the incremental cost-effect pairs indicates that there is little uncertainty regarding the existence of cost-savings with rate-control strategies (in comparison with rhythm-control strategies) because all points fall below the horizontal axis. However, the spread of the points in the vertical plane indicates that there is some uncertainty regarding the magnitude of the cost-savings (-$1,100 to -$11,006). With regard to effectiveness, the location and spread of the points indicate that there is uncertainty regarding the existence and extent of a survival benefit associated with rate-control compared to rhythm-control (from -0.1 years to 0.24 years). This is consistent with the finding of a non-significant difference in survival gain between the two treatment groups. This scatter plot provides additional information about the uncertainty surrounding cost and effects for rate-control versus rhythm-control treatment. However, while these independent incremental cost-effect pairs provide some information about the joint uncertainty in the estimate of incremental cost-effectiveness it is not clear how to present a summary measure of this uncertainty.

#### Uncertainty in cost-effectiveness

There remains considerable debate concerning the presentation of joint uncertainty for estimates of cost-effectiveness. This is partly because there are additional statistical complexities associated with calculating confidence intervals for ratios such as the ICER. As a result of these challenges, a number of alternative methods for calculating confidence intervals have been proposed. These methods include the use of Fieller's theorem and non-parametric bootstrapping [18,19]. However, these solutions do not resolve the problems associated with a small or non-existent effect difference which would cause the ICER to be undefined and may make the variance intractable [20-22]. Furthermore, the appropriate role of uncertainty in the context of decision-making is unclear [13].

Determining the uncertainty surrounding cost-effectiveness requires the investigation of the joint distribution of costs and effects. For the AFFIRM study (Figure 2) the majority of incremental cost-effect pairs fall in the southeast quadrant of the incremental cost-effectiveness plane, indicating that the rate-control strategy is less costly and more effective than the rhythm-control strategy for the management of patients with atrial fibrillation. However, a proportion (approximately 5%) of the points lie in the southwest quadrant, indicating that the rate-control strategy is less costly and also less effective than the rhythm-control strategy. This confirms that there is some uncertainty concerning whether and at what value the rate-control strategy is cost-effective.

### Cost-effectiveness acceptability curves (CEAC)

Faced with the choice of whether or not to reimburse a new technology, the decision maker will likely be interested in the probability that the new technology is cost-effective compared to the existing alternative. This probability can be identified from the incremental cost-effectiveness plane with reference to the decision-maker's defined maximum acceptable ceiling ratio (λ). This probability is simply the proportion of the scatter plot points that fall to the south and east of a ray with slope of λ drawn through the origin (i.e., proportion of incremental cost-effect pairs with a value below λ). Since the maximum acceptable ceiling ratio will generally not be stated explicitly, a sensitivity analysis should be undertaken with the probability determined for a range of λ s. The CEAC provides a plot of these probabilities (y-axis) against λ (x-axis) [23,24].

Cost-effectiveness acceptability curves were introduced as an alternative to calculating confidence intervals for ICERs with statistical methods [23,25]. The CEAC indicates the probability that an intervention is cost-effective compared with the alternative, given the observed data, for a range of λ values. This definition involves a Bayesian definition of probability i.e. the probability that the hypothesis is true ('rate-control strategy is cost-effective') given the data, although the CEAC can be given a Frequentist interpretation [21,22].

**Figure 3:**
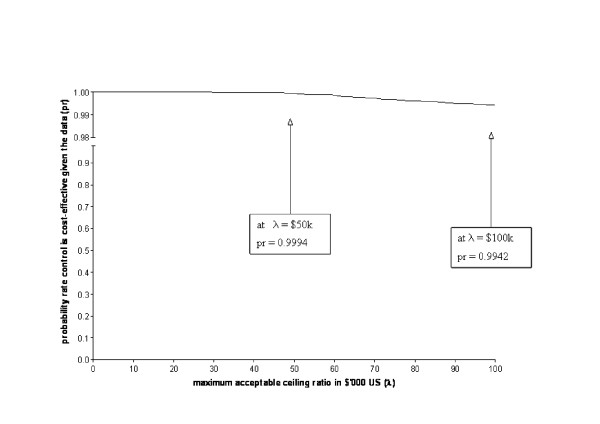
Cost-effectiveness acceptability curve showing the probability that rate-control is cost-effective compared to rhythm-control over a range of values for the maximum acceptable ceiling ratio (λ) in the AFFIRM trial.

Figure 3 shows the CEAC for the probability that a rate-control strategy is cost-effective compared with rhythm-control, for a range of λ s that a decision maker might consider as the maximum cost they are willing to pay for a gain in one year of life. Given a specified value of this 'acceptable' cost-effectiveness ratio (i.e., λ on the x-axis), the CEAC shows the probability (read off on the y-axis) that the data are consistent with a true cost-effectiveness ratio falling below that value.

#### Constructing a CEAC

The CEAC is derived from the joint distribution of incremental costs and incremental effects. When analysing a clinical trial, this distribution is most commonly estimated using non-parametric bootstrapping however it can be generated from a probabilistic analysis of a decision model which translates the uncertainty in input parameters into uncertainty in costs and effects [23,24,26]. The curve is constructed by plotting the proportion of the incremental cost-effect pairs that are cost-effective for a range of λ values. This proportion is identifiable from the incremental cost-effectiveness plane as the proportion of incremental cost-effect pairs lying to the south and east of a ray through the origin with a slope equivalent to the x-axis (i.e., λ = 0). This is repeated for larger and larger λ s until infinity is reached (i.e., the slope of the line is equivalent to the y-axis). Incremental cost-effect pairs that fall in the northwest quadrant are never considered cost-effective and therefore are never counted in the numerator of the estimate. Incremental cost-effect pairs that fall in the southeast quadrant are always considered cost-effective and therefore are always counted in the numerator of the estimate. As λ increases from zero to infinity, incremental cost-effect pairs in the northeast and southwest quadrants may or may not be considered cost-effective (and therefore included in the numerator) depending upon the maximum amount the decision maker is willing to pay for an additional year of life (i.e., λ). As a result, the CEAC does not represent a cumulative distribution function and its shape will depend solely upon the location of the incremental cost-effect pairs within the incremental cost-effectiveness plane (for more details see Fenwick *et al*, 2004 [26]).

#### AFFIRM

For the AFFIRM study, all of the 10,000 bootstrap re-samples involved cost-savings (i.e., fell below the x-axis), hence 100% of the incremental cost-effect pairs fell to the south of the line when λ was zero (i.e., fell below the x-axis); as a result the CEAC crosses the y-axis at 1. When λ was $50,000 per life year gained, a proportion of the re-samples falling within the southwest quadrant are no longer considered cost-effective and therefore are no longer included in the numerator. As a result the proportion of the re-samples that were cost-effective (i.e., fell to the south and east of a ray of slope equal to $50,000 per life year gained) was found to be 99.94%, and when λ equaled $100,000 per life year gained, the proportion was 99.42% (Figure 3). The proportion of the re-samples falling in the northeast or southeast quadrants (i.e. were cost-effective when λ was infinite) was found to be 98.3%. Hence as λ tends to infinity the probability that rate-control is cost-effective compared to rhythm control tends to 0.983 (Figure 3).

#### Interpreting and using CEACs

As stated above, the CEAC indicates the probability that the intervention is cost-effective compared with the alternative, given the data, for a range of values of the maximum acceptable ceiling ratio. Thus, the interpretation of the AFFIRM study is that, given a maximum acceptable ceiling ratio of $50,000 per life year gained, the probability that rate-control is cost-effective compared to rhythm-control is 0.9994. This is equivalent to stating that, given the data, there is a 99.94% chance that the additional cost of rate-control, compared with rhythm-control, is at or below $50,000 per life year gained. Now we have summary measure of joint uncertainty in the estimate of incremental cost-effectiveness.

The information provided by the CEAC is useful to a decision maker faced with the choice of whether or not to adopt a technology because it provides a measure of the uncertainty surrounding the choice (decision uncertainty) given the decision makers selected value of λ. In the case of the AFFIRM study, there was no statistically significant difference in effectiveness between rate-control and rhythm-control strategies for the management of patients with atrial fibrillation. However, the CEAC shows that the decision uncertainty surrounding the adoption of rate-control strategies is less than 1.67% regardless of the maximum acceptable ceiling ratio. Thus, there is very little uncertainty that the decision to adopt rate-control compared to rhythm-control for patients with atrial fibrillation is correct from a cost-effectiveness point of view. It must be noted that statements concerning CEACs should be restricted to those regarding the uncertainty of the estimate of cost-effectiveness. The information from a CEAC *should not*, in general, be used to make statements about the implementation of the intervention (for more details see Fenwick *et al*, 2001 [13]).

## Conclusion

In clinical practice, physicians are increasingly being asked to consider the economic consequences of their treatment decisions. Cost-effectiveness acceptability curves are useful for this purpose because they explicitly incorporate the joint uncertainty – in the effectiveness and cost – into the decision. Furthermore, researchers need to present the consequences of clinical interventions in terms of health benefits and costs to facilitate reimbursement decisions by policy makers. The graphic representation using CEACs facilitates straightforward interpretation by persons unfamiliar with economic evaluation.

In this article, we present the CEAC for a trial of rate-control versus rhythm-control in atrial fibrillation and show how incorporating joint uncertainty affects the interpretation of the results. Specifically, the randomized trial showed no statistical difference in efficacy between rhythm- and rate-control [27], with a trend towards greater benefit in the rate-control arm. The point estimate from a standard cost-effectiveness analysis showed that rate-control was less costly, suggesting that it was the preferred treatment option among patients similar to those enrolled in AFFIRM [12]. The CEAC shown in Figure 3 quantifies the uncertainty in a graphic format. This figure makes it clear that a decision maker can opt for rate-control as the preferred treatment option with a high degree of confidence under a wide range of funding thresholds.

In summary, CEACs are straightforward to calculate, construct, and interpret in addition to having methodological advantages. As a result, they are increasingly being reported in economic evaluations of treatments and will receive greater attention by clinicians, decision-makers, and researchers.

## Competing interests

The author(s) declare that they have no competing interests.

## Authors' contributions

EF – 1) made substantial contributions to conception and design, analysis and interpretation of data; 2) was involved in drafting the manuscript; and 3) has given final approval of the version to be published.

DM – 1) made substantial contributions to conception and design; 2) was involved in revising the manuscript critically for important intellectual content; and 3) has given final approval of the version to be published.

AL – 1) made substantial contributions to conception and design; 2) was involved in revising the manuscript critically for important intellectual content; and 3) has given final approval of the version to be published.

GN – 1) made substantial contributions to conception and design; and 2) has given final approval of the version to be published.

## Pre-publication history

The pre-publication history for this paper can be accessed here:
